# Multi-target synergistic mechanisms of flavonoid compounds from traditional Chinese medicine in non-alcoholic fatty liver disease: insights for human and veterinary medicine

**DOI:** 10.3389/fvets.2026.1804773

**Published:** 2026-05-27

**Authors:** Yu Wang, Caiyi Wan, Lanxin Mu, Aijun Zhu, Ying Li, Yaping Liu, Yadong Bao, Fuxiang Bao

**Affiliations:** 1 College of Veterinary Medicine, Inner Mongolia Agricultural University, Hohhot, China; 2 Inner Mongolia Puze Animal Health Biotechnology Co., Ltd., Hohhot, China

**Keywords:** animal models, flavonoids, lipid metabolism, multi-target synergistic mechanism, non-alcoholic fatty liver disease, traditional Chinese medicine, veterinary medicine

## Abstract

**Introduction:**

Non-alcoholic fatty liver disease (NAFLD) is a prevalent chronic liver disorder characterized by dysregulated hepatic lipid metabolism, with a continuously rising global incidence and limited safe and effective therapeutic options. Importantly, NAFLD-like conditions, namely hepatic lipidosis or fatty liver syndrome, also prevail in veterinary clinical practice, affecting companion animals (obese cats and dogs) and livestock (periparturient dairy cows, fattening pigs, and broiler chickens). These metabolic liver disorders are primarily induced by inappropriate feeding management and metabolic stress, leading to reduced production performance and survival rate of animals, huge economic losses to the livestock industry, and impaired health of companion animals. Flavonoid compounds derived from traditional Chinese medicine (TCM) possess the advantages of low toxicity and multi-target pharmacological effects, and have emerged as promising natural agents for NAFLD amelioration.

**Methods:**

This study adopted a systematic review approach to comprehensively collect, sort out, and summarize recent relevant research findings. We focused on widely reported TCM flavonoids, including quercetin, apigenin, and luteolin, and systematically analyzed the diverse molecular pathways and potential mechanisms by which these compounds exert protective effects against NAFLD and veterinary fatty liver diseases.

**Results:**

Existing research evidence demonstrates that TCM flavonoids improve NAFLD through multiple core regulatory pathways. These compounds reverse hepatic lipid metabolism disorders by activating the *AMPK/SIRT1* and *PPARα* signaling pathways, inhibit lipogenesis by suppressing the key lipogenic factor *SREBP-1c*, and accelerate lipid catabolism by promoting fatty acid *β*-oxidation. In addition, flavonoids effectively alleviate hepatic oxidative stress, inhibit inflammatory responses, and delay the progression of liver fibrosis. Furthermore, they exert protective effects via regulating novel mechanisms, including cellular autophagy, ferroptosis, and intestinal microbiota homeostasis.

**Discussion:**

The multi-target and systematic regulatory characteristics of TCM flavonoids make them excellent candidate natural drugs for NAFLD intervention in both human and veterinary medicine. Nevertheless, several limitations and challenges remain in current research, including low bioavailability of flavonoids and unclear synergistic effects among different flavonoid components. Future studies should focus on improving the bioavailability of flavonoids, elucidating their synergistic molecular mechanisms, and exploring species-specific pharmacokinetic characteristics in cats, dogs, and cattle. Moreover, the development of practical and palatable preparations such as feed additives is essential to promote the clinical translation and large-scale application of flavonoids for the prevention and treatment of NAFLD in human and veterinary clinical practice.

## 1 Introduction

**Figure 1:**
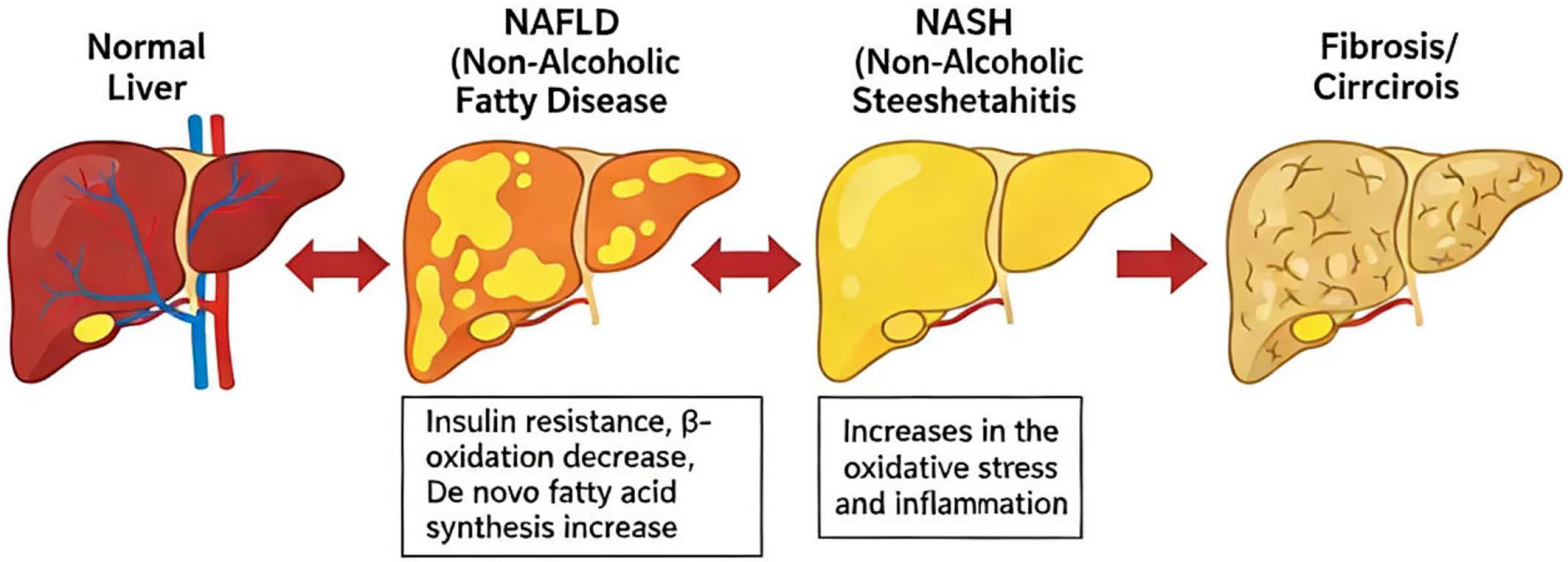
The development of NAFLD.

The liver serves as the central organ for maintaining metabolic homeostasis and detoxification. It plays a critical role in the biotransformation of xenobiotics (1). Non-alcoholic fatty liver disease (NAFLD) is a chronic metabolic disorder closely associated with insulin resistance and metabolic dysregulation, characterized by abnormal accumulation of triglycerides within hepatocytes in the absence of excessive alcohol consumption or other identifiable causes of liver injury (2, 3). The spectrum of NAFLD ranges from simple hepatic steatosis to non-alcoholic steatohepatitis (NASH), hepatic fibrosis, cirrhosis, and may even progress to hepatocellular carcinoma (HCC) (Figure 1) (4). Its primary pathological feature is chronic macrovesicular steatosis, resulting from the accumulation of triglyceride (TG)-laden lipids in hepatocytes (5). Concurrently, evidence of cellular injury, such as ballooning degeneration, apoptotic changes, and characteristic Mallory-Denk bodies is observed, while portal and lobular inflammatory infiltration is more specific to the NASH stage (6).

In recent years, the global incidence of NAFLD has increased rapidly with improvements in quality of life. As liver disease progresses relatively slowly and carries risks of end-stage hepatic fibrosis, cirrhosis, and ultimately hepatocellular carcinoma, controlling and treating this disease should be prioritized (7, 8). The goal of NAFLD treatment is to improve or stabilize liver histological damage, slow fibrosis progression, prevent mortality associated with adverse liver-related outcomes, and prevent worsening of related cardiometabolic comorbidities (9). NAFLD, as a chronic metabolic disease, may require slow therapeutic progress; therefore, any pharmacological intervention must not only be effective but also exhibit adequate safety and tolerability. Currently, the cornerstone of NAFLD management remains a healthy lifestyle intervention. Available pharmacological options remain limited, often failing to fully address the complex pathogenesis of the disease and potentially carrying side effects. For example, obeticholic acid (OCA) has shown potential in clinical trials to improve NASH histology (approximately 45% of patients achieved a ≥ 2-point reduction in NASH score without worsening fibrosis), but its use is limited by adverse effects such as pruritus (9). In this context, natural products have attracted widespread attention due to their multi-target regulatory advantages.

Traditional Chinese medicine (TCM), known for its high safety profile, minimal side effects, and the characteristics of “multiple components, multiple targets, and multiple pathways,” may offer benefits in reversing fatty liver with sustained use (3). Flavonoids are a class of natural polyphenolic compounds found in various plants with broad pharmacological activities and favorable therapeutic effects. They exhibit multiple functions, including antioxidant, anti-inflammatory, and immunomodulatory effects, and offer advantages such as low toxicity and low risk of inducing drug resistance (7). Growing evidence indicates that flavones, flavonols, isoflavones, and flavanones can exert protective effects in NAFLD by activating the *AMPK/SIRT1* pathway, inhibiting the *NF-κB/NLRP3* inflammasome, improving insulin sensitivity, and modulating the gut-liver axis. These mechanisms make flavonoids a current research focus for treating NAFLD (5). This article systematically reviews the pharmacological effects and molecular mechanisms of flavonoids in intervening in NAFLD in recent years, aiming to provide a theoretical basis for developing multi-target therapeutic strategies based on natural products.

While the pathophysiological mechanisms of NAFLD have been extensively elucidated in humans, accumulating evidence highlights its considerable prevalence and profound clinical implications in veterinary species. Among companion animals, feline hepatic lipidosis, a life-threatening form of fatty liver disease predominantly triggered by anorexia in obese cats, shares pivotal metabolic derangements and oxidative stress-related pathological features with human NAFLD/NASH (10). Similarly, canine obesity, an increasingly prominent animal welfare concern globally, is being progressively linked to hepatic steatosis and inflammatory responses (11). Beyond companion animals, metabolic-associated fatty liver disease poses substantial challenges to livestock production systems worldwide. Dairy cows are particularly vulnerable to this condition during the periparturient period, as negative energy balance induces excessive adipose tissue mobilization and subsequent hepatic lipid accumulation. This pathological process not only impairs milk production and reproductive performance, but also compromises overall herd health and sustainability (12, 13). Analogously, broiler chickens genetically selected for rapid growth rates frequently develop fatty liver hemorrhagic syndrome (FLHS), which results in significant economic losses to the poultry industry and severe animal welfare implications (14, 15). Despite these striking pathological and etiological parallels with human NAFLD, therapeutic options for veterinary NAFLD remain highly limited and primarily confined to supportive care. Thus, exploring natural, multi-target bioactive agents, such as flavonoid compounds derived from traditional Chinese medicine (TCM), represents a promising avenue not only for advancing human NAFLD therapeutics but also for developing safe, effective, and species-tailored interventions in veterinary medicine. While anchored in mechanistic insights from human and rodent model studies, this review aims to establish a foundational knowledge framework that can guide future translational research and facilitate the clinical application of these interventions across diverse animal species.

## 2 Correcting hepatic glucose and lipid metabolism disorders

Metabolic dysfunction leads to hyperglycemia and dyslipidemia, which can promote hepatic steatosis and exacerbate the development of NAFLD (15). Insulin resistance impairs glucose homeostasis by disrupting regulatory pathways involved in cellular glucose uptake, leading to impaired glucose metabolism. This imbalance between energy intake and expenditure further promotes hepatic steatosis, contributing to abnormal lipid metabolism in NAFLD (16). Therefore, correcting disturbances in hepatic glucose and lipid metabolism to restore metabolic homeostasis is essential for the treatment and improvement of NAFLD.

### 2.1 Regulating hepatic lipid metabolism

Hepatic lipid metabolism disorder is central to the pathogenesis of NAFLD. Under normal physiological conditions, the liver regulates lipid synthesis and degradation through multiple mechanisms (17). Flavonoids possess antibacterial, antiviral, and anti-inflammatory properties, which can indirectly influence hepatic lipid metabolism, as well as directly regulate fat metabolism. Quercetin, widely present in photosynthetic plants, is currently a major research focus as the most abundant dietary natural flavonoid compound (18). By activating the *FXR1/TGR5* signaling pathway, it reduces lipid accumulation, inflammation, and oxidative stress in hepatocytes both *in vivo* and *in vitro* (1), it also promotes hepatic very-low-density lipoprotein assembly and lipophagy via the *IRE1α/XBP1s* pathway (19). AMPK serves as a cellular energy sensor; activation of hepatic *AMPK* alleviates NAFLD through promoting fatty acid oxidation, inhibiting hepatic lipogenesis and cholesterol synthesis, regulating liver energy metabolism, reducing the activity of enzymes related to intrahepatic fat storage, and decreasing triglyceride accumulation (20). Apigenin not only increases *AMPK* levels and reduces the expression of *HMGCR, SREBP-1, FAS*, and S*REBP-2*, but also decreases hepatic fat accumulation and improves lipid metabolism by activating the *AMPK/SREBP* signaling pathway (20). Furthermore, kaempferol (*KMP*) reduces lipogenesis and promotes fatty acid oxidation by activating the *Sirt1/AMPK* pathway. Additionally, activation of Sirt1/AMPK enhances the expression of proliferator-activated receptor gamma coactivator 1-alpha (*Pgc1α*), which in turn activates mitochondrial fatty acid oxidation and promotes fatty acid excretion (21).

Using an oleic acid-induced HepG2 cell model of NAFLD, it was found that puerarin activates the *PPARα-AMPK* signaling pathway. This leads to a reduction in the expression levels of lipogenic enzymes such as *FAS* and *SREBPs*, while increasing the expression level of *PPARα*, thereby inhibiting lipogenesis and enhancing antioxidant activity. These findings suggest that puerarin extracts may possess therapeutic value in treating fatty liver and lipid-related metabolic disorders (22). Resveratrol inhibits fat synthesis and exerts liver-protective and lipid-lowering effects by upregulating the expression of fatty acid *β*-oxidation factors (*PON1, PPAR-α*) and downregulating the expression of lipogenic factors (*Fdft1, FASN, PPAR-γ*) (23). In addition, luteolin can prevent obesity and improve hepatic steatosis by regulating the liver X receptor (*LXR*)-*SREBP-1c* signaling pathway to inhibit hepatic lipogenesis and by increasing biliary cholesterol excretion (24, 25). Citrus fruits are rich in various flavonoids (such as naringin and hesperetin). These compounds can activate the *AMPK* signaling pathway and modulate transcription factors like *PPARα* and their downstream targets (e.g., *CPT1, SREBP-1c*), thereby enhancing fatty acid oxidation and suppressing hepatic fatty acid synthesis. Consequently, they effectively ameliorate hepatic steatosis and dyslipidemia (22, 98). Common flavonoids exert anti-NAFLD effects through multiple dimensions, such as regulating lipid metabolism, inhibiting lipogenesis, and alleviating hepatic steatosis, by targeting specific signaling pathways or key factors including *FXR1/TGR5* and *AMPK/SREBP*. The specific corresponding relationships are summarized in Table 1.

**Table 1:** Molecular targets and functional roles of flavonoids in lipid metabolism regulation.

Flavonoids	Target signaling pathways/key factors	Specific function and effect
Quercetin	FXR1/TGR5, IRE1a/XBP1s	Alleviating lipid accumulation, inflammation, and oxidative stress; promoting VLDL assembly and lipophagy (5, 18)
Apigenin	AMPK/SREBP	Reducing hepatic steatosis and improve lipid metabolism (5)
kaempferol (KMP)	Sirt1/AMPK	Reducing hepatic fat synthesis, promote fatty acid oxidation, and activate PGC-1α (20)
Puerarin	PPARα-AMPK	Reducing the expression of lipogenic enzymes and inhibit lipogenesis, while enhancing PPARα activity and boosting antioxidant capacity (21)
Resveratrol	PON1, PPAR-α; Fdft1, FASN, PPAR-γ	upregulating fatty acid β-oxidation factors, downregulate lipogenic factors, and inhibit de novo lipogenesis (22)
Luteolin	LXR-SREBP-1c	Suppressing hepatic lipogenesis and enhance biliary cholesterol excretion, thereby preventing obesity and ameliorating hepatic steatosis (23, 24)
Citrus flavonoids (e.g., naringin, hesperetin)	transcription factors such as AMPK and PPARα, and their downstream targets (CPT1, SREBP-1c)	Enhancing fatty acid oxidation, inhibit fatty acid synthesis, and regulate hepatic steatosis and dyslipidemia (25)

### 2.2 Regulating hepatic glucose metabolism

Numerous studies have demonstrated that insulin resistance is closely associated with the development of NAFLD (26). This core issue lies in the fact that insulin resistance impairs glucose uptake by peripheral tissues, such as skeletal muscle and adipose tissue, which promotes lipolysis, leading to increased circulating lipid levels, thereby further suppressing the anti-lipolytic effect of insulin (27). Consequently, ameliorating insulin resistance may delay the progression of NAFLD. Therefore, the progression of NAFLD may be attenuated by improving insulin resistance. Anthocyanins in mulberry can increase the phosphorylation level of hepatic *AMPK* and reduce the levels of *G6Pase* and *PEPCK*, thereby inhibiting hepatic gluconeogenesis (19). Silymarin plays a role in modulating metabolic processes, protecting the liver and improving hepatic tissue conditions by reducing total cholesterol, triglycerides, and low-density lipoprotein cholesterol (LDL-C), and increasing high-density lipoprotein cholesterol (HDL-C, the “good” cholesterol), as well as reducing fasting insulin levels and improving insulin resistance (28). The total flavonoids from *Albizia julibrissin* exhibit glucose and lipid metabolism regulatory activity. At low concentrations, total flavonoids from *Albizia julibrissin* exhibits a mild inhibitory effect on obesity induced by metabolic disorders, and at higher concentrations of the total flavonoids from *Albizia julibrissin* primarily target the correction of glucose metabolism disorders to Enhancing the signaling efficiency of the *PI3K/akt* and *AMPK* pathways and regulatory effects of metabolic function-related proteins can exert regulatory effects on glucose metabolism (29). The flavonoid kaempferol from *Coreopsis tinctoria* reduces body weight and blood glucose, improves glucose tolerance, and ameliorates insulin resistance in obese mice by activating the IRS-1/PI3K/akt insulin signaling pathway and downregulating the expression of *PEPCK* and *G6Pase* (30). Hawthorn leaf total flavonoids effectively inhibit pancreatic islet cell apoptosis, thereby increasing insulin secretion, lowering blood glucose levels, and reducing oxidative stress-induced damage (26). In summary, flavonoids help protect the liver by improving insulin resistance and modulating hepatic glucose metabolism toward homeostasis, thus providing an important theoretical basis for nutritional intervention and drug development in NAFLD.

In veterinary species, flavonoids exert conserved yet species-specific lipid-regulating effects. Quercetin administered intraduodenally improves hepatic lipid deposition in periparturient dairy cows via *AMPK/PPARα* activation (31). In cats with spontaneous hepatic lipidosis, apigenin and luteolin suppress SREBP-1c-mediated lipogenesis, but feline glucuronidation deficiency markedly reduces bioavailability (32). In broiler chickens, citrus flavonoids (naringin, hesperetin) alleviate fatty liver hemorrhagic syndrome (FLHS) through *AMPK* signaling (14). These differences arise from distinct digestive anatomy, metabolic enzyme profiles, and pharmacokinetic traits across cats, dogs, cattle, and poultry.

## 3 Attenuating oxidative stress

In the development and progression of NAFLD, oxidative stress plays a critical role. Hepatic steatosis promotes excessive production of reactive oxygen species (ROS), which in turn induces mitochondrial dysfunction and endoplasmic reticulum stress, leading to further ROS generation and forming a vicious cycle (33). This process not only impairs mitochondrial respiratory function and drives the progression from simple steatosis to NASH, but also causes peroxisomal dysfunction, exacerbating inflammatory responses, DNA damage, and pro-fibrotic signaling, thereby continuously aggravating hepatocellular injury (34). Therefore, ameliorating oxidative stress is of significant importance in slowing the progression of NAFLD.

### 3.1 Modulating relevant factors

Flavonoids may suppress oxidative stress by modulating factors such as malondialdehyde (MDA), superoxide dismutase (SOD), and catalase (CAT) (7). Total flavonoids from *Broussonetia papyrifera* leaves (TFBP) not only effectively inhibit the generation of reactive oxygen species (ROS), enhance SOD activity, and reducing myeloperoxidase levels to alleviate oxidative stress injury, but also regulating oxidative stress through the *Nrf2/HO-1* signaling pathway, promoting nuclear translocation of *Nrf2* and increasing *HO-1* expression, thereby improving antioxidant capacity (7). In hyperlipidemic mice and NASH cell models, *α*-naphthoflavone (ANF) reduced harmful ROS and MDA levels while elevating the activities of antioxidant enzymes including SOD, CAT, and glutathione (GSH), and effectively alleviating oxidative stress in hepatocytes (35). Dandelion contains various active components such as flavonoids and polyphenols. Feeding NAFLD model mice with dandelion extract resulted in significantly reduced levels of T-SOD, GSH-Px, and CAT in liver tissue, suggesting its potential to ameliorate NAFLD pathology by regulating oxidative stress (35, 99). Different polar extracts of total flavonoids from Scutellaria amoena improved symptoms in NAFLD model rats by modulating oxidative stress and inhibiting inflammatory factor secretion, with the n-butanol extract showing the most pronounced effect (36).

### 3.2 Modulating related signaling pathways

The functional role of these signaling pathways is to maintain hepatic redox homeostasis, suppress oxidative damage, and prevent the progression from simple steatosis to NASH*. Nrf2* is an oxidative stress-mediated transcription factor, and its deficiency disrupts redox homeostasis, exacerbates oxidative damage to membranes and organelles, and accelerates the transition from simple steatosis to NASH (37). Hesperetin alleviates hepatic oxidative stress via the *PI3K/akt-Nrf2* pathway, and further triggers the *PI3K-akt-Nrf2-SOD/GPx/GCLC/GR/HO-1* signaling cascade, whose function is to neutralize excessive ROS, reduce lipid peroxidation, and protect hepatocytes from oxidative injury. Additionally, it inhibits fatty acid-induced *NF-κB* activation and subsequent inflammation by lowering ROS overproduction through the *Nrf2* signaling cascade (38). *MAPKs* and *Nrf2* play regulatory roles in anthocyanin-mediated activation of antioxidant enzymes. Further studies indicate that, in combination with troglitazone, anthocyanins can activate the *PPARγ-Nrf2* signaling pathway, whose function is to ameliorate hydrogen peroxide-induced dysregulation of lipid metabolism-related gene expression (39). Ponkan peel flavonoids (PTFC) exhibit anti-inflammatory, antioxidant, and intestinal mucosal barrier repair functions. Network pharmacology-based predictions of their targets suggest that PTFC alleviates oxidative stress damage in NAFLD by promoting *Nrf2* nuclear translocation, activating the *Nrf2/ARE*‌ antioxidant signaling pathway, whose function is to upregulate a series of phase II detoxification enzymes and antioxidant enzymes (40). Saikosaponin B1, a major active component in Bupleurum, demonstrates potential as a candidate drug for liver disease treatment by enhancing SOD activity, reducing the production of lipid peroxidation product MDA, suppressing inflammatory responses, and mitigating oxidative stress through activating the *Nrf2/HO-1* pathway (41). Quercetin reduces oxidative stress in NASH rats by inhibiting *NLRP3* inflammasome activation, lowering total ROS and H₂O₂ levels, enhancing SOD activity, and suppressing the ROS/thioredoxin-interacting protein (TXNIP) pathway (42). Administration of different doses of *Cyrtomium fortunei* flavonoids to high-fat diet model mice for four weeks revealed that these flavonoids improve lipid metabolism disorders, reduce inflammatory responses and oxidative stress by modulating the *AMPK/Sirt1/NF-κB* pathway (43). In summary, flavonoids alleviate oxidative stress by functionally regulating these signaling pathways to enhance antioxidant capacity, reduce ROS accumulation, and protect hepatocytes, thereby delaying and ameliorating NAFLD.

Veterinary studies confirm the *Nrf2/HO-1* pathway is a conserved target across species. In cats with hepatic lipidosis, quercetin and baicalin reduce MDA and elevate SOD/CAT activities but require low starting doses due to feline oxidative sensitivity (10). In transition dairy cows, TCM flavonoids mitigate oxidative stress linked to negative energy balance (44). In dogs with obesity-related hepatopathy, green tea polyphenols and dandelion flavonoids improve redox status (45). Felines show limited glucuronidation capacity, leading to prolonged flavonoid exposure and narrower safety margins.

## 4 Combating inflammatory response

### 4.1 The NF-κB and NLRP3 signaling pathways

NLRP3 inflammasome exacerbates the inflammatory response in NAFLD, primarily activated by lipid metabolites such as TG and FFAs (32). Subsequently, *NF-κB* drives the transcriptional upregulation of *NLRP3*, promoting inflammasome assembly. Activated Caspase-1 activity then cleaves *pro-IL-1β* and *pro-IL-18* into their mature, bioactive forms, triggering sustained inflammation and leading to hepatocyte apoptosis and necrosis (46). Inhibition of the *NF-κB* pathway has been shown to ameliorate NAFLD progression. Calycosin glucoside significantly down-regulates the expression of *p-NF-κB/NF-κB* and increases the expression of *PPAR-γ* and *Nrf2* at both protein and gene levels, suggesting its evident regulatory role in the *PPAR-γ/NF-κB* pathway (47). Supplementation with epigallocatechin gallate (EGCG) in a diet-induced rat model of exacerbated liver injury significantly improved glutathione (GSH) status, thereby suppressing hepatic and adipose inflammation mediated via the *NF-κB* pathway (48). Furthermore, silymarin stimulates the production of endogenous antioxidants such as GSH and alleviates inflammation by inhibiting *NF-κB* and reducing *TNF-α* levels (49). Kaempferol prevents the ubiquitination and degradation of IκB, increases IκB levels in cells and rat liver, consequently inhibiting *NF-κB* nuclear translocation and elevating cytoplasmic levels of *NF-κB* and *p-NF-κB*. This further reduces the expression of *TNF-α* and *IL-6* to ameliorating hepatic steatosis and liver fibrosis (50). Quercetin effectively upregulates *PPAR-α* expression by modulating the hepatic *NF-κB* pathway in NAFLD mice, thereby inhibiting *p-IκBα* and preventing *IκBα* ubiquitination and degradation. This subsequently suppresses *NF-κB* expression, downregulates MDA levels, increases SOD and CAT expression, attenuates oxidative stress, and alleviates hepatic lipotoxicity (51). Treatment of high-fat diet model mice with trilobatin in combination with streptozotocin (STZ) revealed that trilobatin not only restored glucose metabolic disorders and liver function in diabetic mice, alleviated hepatic steatosis and liver fibrosis, as well as mitigated liver injury, but also downregulated the expression levels of the *NLRP3* inflammasome, *NF-κB p65*, and activated *Caspase-1* and G*SDMD-N*. Furthermore, it inhibited the release of *IL-18* and *IL-1β*, thereby reducing hepatic inflammatory response and hepatocyte pyroptosis (52). Icaritin (ICA) significantly decreases serum levels of ALT and AST, increases HDL-C levels, improves liver tissue damage and lipid deposition, reduces *IL-1β*, *IL-12*, and *IL-6* levels in liver tissue and cells, and suppresses the expression of proteins related to the MAPK and *NF-κB* signaling pathways in liver tissue (53).

Flavonoids also ameliorate NAFLD by inhibiting the *NLRP3* inflammasome. As a key mediator of pyroptosis, *NLRP3* activates Caspase-1, which promotes the expression of the inflammatory cytokines *IL-1β* and *IL-18* and cleaves GSDMD, leading to the release of its active N-terminal domain (54). Beyond suppressing hepatic inflammation via the *NF-κB/TLR/NLRP3* signaling pathway, mitigating oxidative stress injury through the *PI3K/Nrf2* pathway, and modulating the activation state of the mTOR pathway during autophagy. Quercetin also suppresses the expression of apoptotic factors closely associated with liver disease progression (55). Baicalin likely alleviates NAFLD through a multicomponent and multitarget mode of action by forming a robust hydrogen bonding network with various amino acid residues of *TLR4* and *NF-κB*, thereby blocking the *TLR4/NF-κB/NLRP3* pathway (56). Naringenin exerts preventive and therapeutic effects on NAFLD by downregulating the expression of the NLRP3/NF-κB signaling pathway in both Kupffer cells (KCs) and hepatocytes, and reducing the levels of *NF-κB*, *NLRP3*, *IL-1β*, and *IL-18*, and thereby inhibiting hepatic inflammation in mice (57). In summary, targeting the *NF-κB* pathway and the *NLRP3* inflammasome using flavonoids holds promising research potential for the treatment of NAFLD, providing novel insights and strategies for NAFLD therapy and drug development.

### 4.2 TLR4 signaling pathway

Toll-like receptors (TLRs) are a family of pattern recognition receptors that play a crucial role in the host immune system. Aberrant activation of the *TLR4* pathway promotes the release of pro-inflammatory cytokines, induces oxidative stress, and exacerbates insulin resistance, thereby accelerating the progression of NAFLD (58). Hesperidin significantly improves liver function and reduces blood lipids, likely achieved by downregulating the expression of *TLR4* and *TLR9*, thereby attenuating the inflammatory response (59). Genistein effectively ameliorates NAFLD by downregulating the expression levels of *TLR4* protein and gene in the liver tissue of NASH model rats, reducing serum endotoxin and hepatic TNF-*α* content, alleviating hepatic inflammation, and simultaneously normalizing various serum and hepatic biochemical parameters (60). Luteolin significantly alters the composition of gut microbiota in NAFLD rats, thereby reducing the secretion of pro-inflammatory factors via inhibition of the hepatic *TLR4* signaling pathway. It also suppresses the activation of the *TLR4* signaling pathway in the liver, decreases the secretion of pro-inflammatory factors, and mitigates the pathological state of NAFLD (61). Thus, *TLR4* plays a key role in the development and progression of NAFLD.

### 4.3 Other relevant mechanisms associated with immune dysfunction

NAFLD progression is closely accompanied by hepatic immune dysfunction, characterized by imbalanced macrophage polarization, disordered T-lymphocyte subsets, and abnormal cytokine secretion (62). Based on their functional states, macrophages can be classified into pro-inflammatory M1 macrophages and anti-inflammatory M2 macrophages. M1 macrophages induce a strong inflammatory response by releasing pro-inflammatory cytokines such as *IL-1β*, *IL-6*, and *TNF-α*, thereby aggravating hepatic immune dysfunction. In contrast, M2 macrophages primarily contribute to tissue repair, wound healing, and the clearance of cellular debris through the secretion of arginase-1 (*Arg-1*), *IL-10*, transforming growth factor-β (*TGF-β*), and matrix metalloproteinases (*MMPs*) (62). M1 macrophages have been identified as the primary effector cells driving excessive hepatic inflammation (63). Baicalin demonstrates significant potential in treating NAFLD by improving immune dysfunction via regulating macrophage polarization and the inflammatory response. CD4^+^ T cells suppress inflammation by regulating the function of adipose tissue macrophages (*ATMs*), whereas CD8^+^T cells promote M1 macrophage polarization. Increasing the CD4/CD8 ratio can enhance immune surveillance, inhibit inflammation, and significantly improve obesity by regulating glucose and lipid metabolism, thereby preventing further hepatic fat accumulation (64).

The *PI3K/akt* signaling pathway plays a critical role in regulating hepatic immune responses and inflammatory injury. Studies utilizing *in vitro* cell experiments, *in vivo* animal models, and ultra-performance liquid chromatography–high-resolution mass spectrometry (UPLC-Q-TOF/MS) combined with network pharmacology and molecular docking techniques have demonstrated that total flavonoids from *Carthamus tinctorius* L. leaves (TFCTLL) possess anti-inflammatory and antioxidant activities. TFCTLL can further enhance its anti-inflammatory effects through the *PI3K/akt* pathway, effectively reduce inflammatory cytokines, and ameliorate pathological liver injury (65).

Flavonoids inhibit *NF-κB/NLRP3* and *TLR4* pathways in veterinary NAFLD models. Luteolin and baicalin suppress hepatic *NLRP3* inflammasome activation in cats with lipidosis (10). Quercetin and naringenin improve canine obesity-induced steatohepatitis via *TLR4/NF-κB* inhibition (45). In laying hens with FLHS, ginkgo flavonoids reduce hepatic inflammation and hemorrhagic lesions (14). Ruminants need rumen-protected formulations; cats lack glucuronidation, requiring cautious dose titration.

## 5 Delaying hepatic fibrosis

During the progression of NAFLD, the onset of fibrosis is generally regarded as a mid-to-late-stage event linking NASH to cirrhosis. Activation of hepatic stellate cells (HSCs) is a key factor in liver fibrosis, leading to excessive deposition of extracellular matrix (ECM) and scar formation (20). Therefore, inhibiting the fibrotic process can prevent the worsening of NAFLD and is of significant clinical importance. Inflammatory responses contribute to fibrosis in normal hepatocytes and accelerate the progression of NAFLD (17). Total flavonoids of *Artemisia annua* L. reduce the expression of pro-inflammatory cytokines *TNF-α*, *IL-1β*, and *IL-6* in local liver tissue and peripheral blood, promote the expression of the anti-inflammatory cytokine *IL-10*, attenuate local inflammatory responses, and alleviate liver injury, thereby exerting therapeutic effects against hepatic fibrosis (66). Consequently, anti-inflammatory intervention also represents an effective approach to suppress liver fibrosis. Research indicates that the anti-fibrotic mechanism of Bupleurum hamiltonii decoction in rats is associated with regulating *MMP-2* and *TIMP-2* expression and reducing excessive ECM deposition (67). Wogonin (Wog) alleviates the degree of liver fibrosis in rat models, likely through inhibition of the Hedgehog-YAP signaling pathway (68). Total flavonoids from litchi seeds inhibit hepatic inflammation, improve liver function, and exert anti-fibrotic effects by suppressing the *TGF-β/Smad* signaling pathway (69). Yin Chen Hao Tang, primarily indicated for yang-type jaundice, contains key active components such as rhein, kaempferol, and quercetin. It inhibits hepatocyte apoptosis by downregulating cleaved caspase-3 and upregulating the expression of *p-ERK1/2*, *PI3K*, and *Bcl-XL*, thereby demonstrating its ability to alleviate hepatic fibrosis (70). Total flavonoids of *Penthorum chinense* Pursh reduce fibrosis markers (*Col-IV*, *PC-III*, *HAase* and *LN*), potentially via the *TLR4/NF-κB* or *TGF-β/Smad* pathways, though further investigation is needed (71).

In summary, flavonoids can delay the occurrence of hepatic fibrosis through modulating specific signaling pathways, reducing excessive ECM deposition, exerting anti-inflammatory effects, and inhibiting hepatocyte apoptosis. However, relying on a single approach to inhibit liver fibrosis often yields suboptimal therapeutic outcomes. Combining herbal monomers into compound formulations and identifying additional therapeutic targets may lead to more efficient treatment strategies.

Anti-fibrotic effects of flavonoids are highly relevant to veterinary chronic liver disease. In dairy cows with fatty liver-associated fibrosis, quercetin and *Artemisia annua* flavonoids inhibit HSC activation and *TGF-β/Smad* signaling (12). In dogs with chronic hepatitis, silymarin and wogonin reduce ECM deposition. Cats rarely progress to advanced fibrosis due to acute lipidosis mortality, but flavonoids still mitigate early fibrogenic signals (10).

## 6 Modulating hepatocyte death and survival

### 6.1 Modulating ferroptosis

Ferroptosis is a novel form of cell death triggered by excessive iron accumulation, leading to iron-dependent lipid peroxidation and elevated levels of reactive oxygen species (ROS). In recent years, numerous studies have demonstrated a close association between ferroptosis and liver diseases. Compared to normal liver tissue, diseased livers exhibit increased levels of iron ions and lipid ROS, suggesting that ferroptosis may play a significant role in hepatic pathologies (72). Ginkgolide B (GB), a major component of *Ginkgo biloba* extract, can specifically ameliorate ferroptosis induced by lipid accumulation and oxidative stress in NAFLD via the *Nrf2* signaling pathway, thereby exerting hepatoprotective effects (73). Exosomes derived from mesenchymal stem cells, after treatment with baicalin, regulate iron homeostasis in hepatocytes by activating the *Keap1-Nrf2* pathway and upregulating p62 expression (74). Total flavonoids of Melastoma dodecandrum Lour. (TFMD) alleviate iron overload and lipid peroxidation accumulation in the liver tissues of NAFLD rats, indicating that the hepatoprotective effect of TFMD is closely related to reducing hepatic lipid peroxide accumulation and modulating iron metabolism imbalance. This is achieved by activating the body’s antioxidant system and regulating the ferroptosis-related *PPARγ/PGC-1α/Nrf2* signaling pathway, thereby delaying the onset and progression of NAFLD (75). Kaempferol demonstrates hepatoprotective effects and stimulates autophagy through the activation of *AMPK*, which is associated with inhibiting ferroptosis related to iron-induced oxidative stress and iron accumulation. Moreover, kaempferol exhibits definite protective effects against both iron-induced oxidative stress and carbon tetrachloride-induced liver injury (76). Flavonoids delay the progression of NAFLD by modulating ferroptosis, offering a new perspective for the treatment of NAFLD.

### 6.2 Inhibiting apoptosis

Apoptosis in hepatocytes is closely associated with oxidative damage and is primarily triggered through three pathways: the mitochondrial apoptosis pathway, the death receptor pathway, and the endoplasmic reticulum stress pathway (77). Puerarin significantly reduces the activity of ALT and AST in rat serum and simultaneously inhibits hepatocyte apoptosis, thereby alleviating liver injury (78). Dihydromyricetin (DMY) protects human normal hepatocyte L02 cells from H_2_O_2_-induced oxidative damage by inhibiting endoplasmic reticulum stress (79). The protective effect of Sinisan on liver function in mice with liver fibrosis significantly ameliorates the degree of hepatic fibrosis, likely achieved by its main active component, isorhamnetin, which inhibits the akt target and upregulates *FXR* expression to improve apoptosis (80). The apoptosis rate induced by *AFB1* is significantly suppressed by Baicalin, with *JNK* potentially serving as a key target for alleviating AFB1-induced hepatocyte apoptosis (81). Luteolin mitigates palmitate-induced hepatocyte injury and apoptosis by inhibiting endoplasmic reticulum stress mediated by the *PERK/eIF2α* pathway and activating autophagy, thereby exerting hepatoprotective effects (82). Flavonoids protect the liver by inhibiting hepatocyte apoptosis, which holds significant importance for the treatment of NAFLD.

### 6.3 Modulation of autophagy-related pathways

Autophagy is a fundamental catabolic process that maintains cellular homeostasis by degrading damaged organelles and misfolded proteins. Dysregulation of autophagy accelerates the development of NAFLD. Meanwhile, ROS generated during oxidative stress can induce mitochondrial dysfunction and subsequently suppress autophagy (83, 84). EGCG has been shown to promote hepatocyte proliferation and autophagy, inhibit mitochondria-dependent apoptosis, and enhance autophagic activity in high-fat diet-fed mice, potentially via the ROS-mediated *MAPK* pathway (85). Quercetin can also inhibit hepatic stellate cell activation and reduce autophagic activity by modulating the crosstalk between the *TGF-β1/Smads* and *PI3K/akt* pathways, ultimately exerting anti-fibrotic effects (86). Galangin, a flavonol derivative of curcumin, can both ameliorate existing hepatic steatosis and prevent its onset by promoting hepatocyte autophagy, thereby exerting preventive and therapeutic effects on liver steatosis. This action is likely associated with the enhancement of hepatocyte autophagy. Furthermore, it reduces intracellular lipid accumulation and elevates autophagic activity in hepatocytes (87). Mangiferin, a natural C-glucosyl flavonoid, exhibits multiple biological activities. It alleviates inflammation by inhibiting *NF-κB* and *JNK* activity, regulates autophagy through the *AMPK/mTOR* signaling pathway, and improves glucose and lipid metabolism by modulating the *IRS/PI3K/akt* pathway. In summary, the modulation of autophagy is a key therapeutic target for NAFLD, as it regulates hepatic lipid metabolism, apoptosis, and inflammatory responses. Further in-depth research into the mechanisms and approaches by which flavonoids regulate autophagy may provide new strategies for the prevention and treatment of NAFLD.

Modulation of ferroptosis, apoptosis, and autophagy differs across veterinary species. In feline hepatic lipidosis, kaempferol and ginkgolide B inhibit ferroptosis via *Nrf2* but are limited by low *GSH* synthesis (10). In broilers with FLHS, baicalin and puerarin reduce hepatocyte apoptosis and enhance autophagy (14). In transition cows, EGCG and quercetin restore autophagic flux to prevent lipotoxicity. Feline poor glucuronidation, rumen degradation, and avian rapid transit strongly impact bioactivity.

## 7 Modulation of gut microbiota

**Figure 2:**
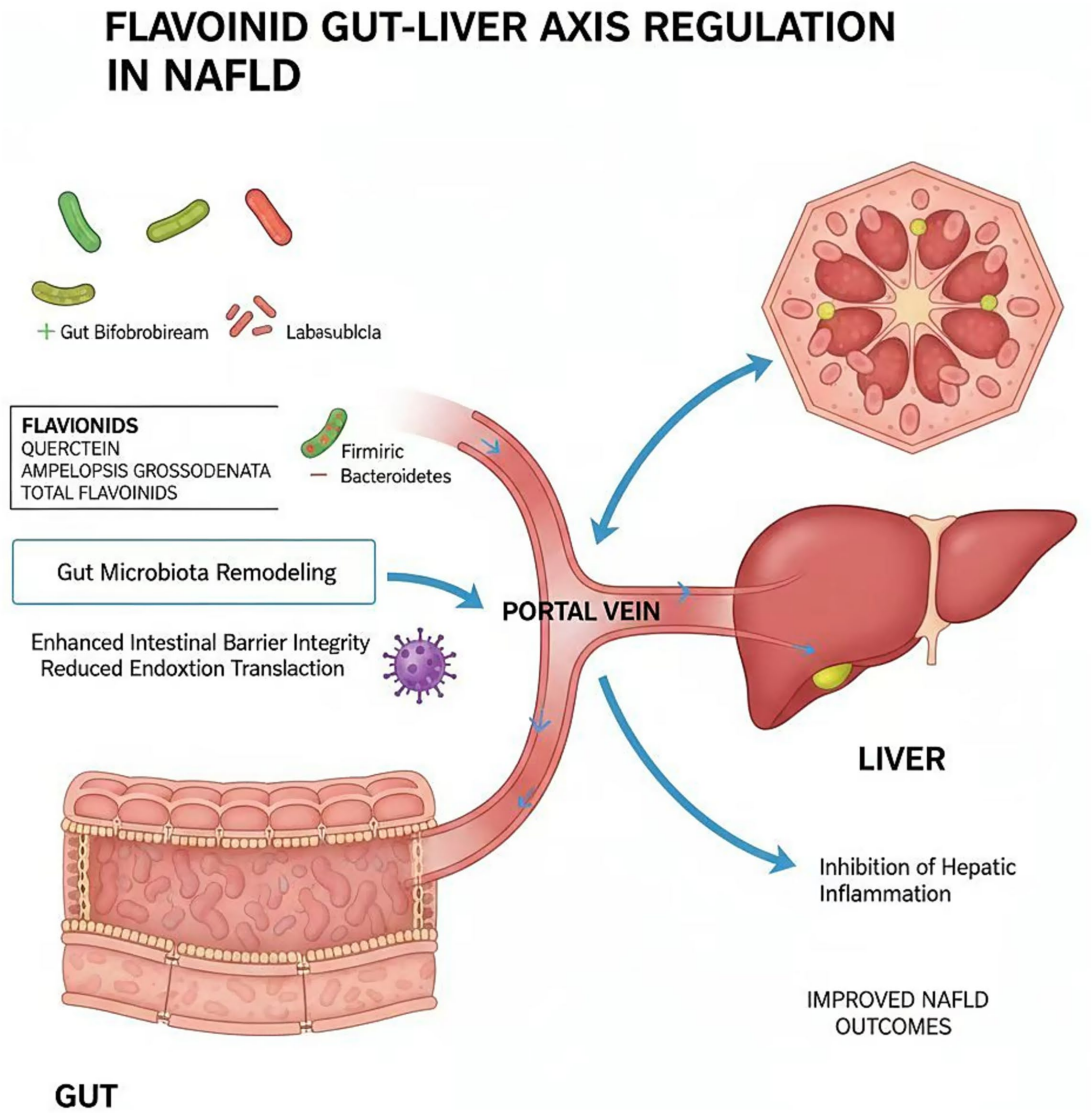
Flavonoids improve NAFLD by regulating the gut-liver axis.

Intestinal dysbiosis may induce hepatic injury. Ethanol produced by certain pathogenic bacteria within the gut, such as *Escherichia coli*, can compromise intestinal barrier integrity. This leads to small intestinal bacterial overgrowth and increased intestinal permeability, ultimately resulting in chronic endotoxemia. Elevated endotoxin translocates from the intestinal lumen into the portal circulation triggering the generation of reactive oxygen species (ROS), which further exacerbates hepatic inflammation. Bacterial overgrowth, disruption of the enterohepatic bile acid circulation, increased endotoxin absorption, and bacterial translocation are all implicated in the pathogenesis and progression of NAFLD. These processes promote the advancement of NAFLD to hepatic fibrosis, NASH and even hepatocellular carcinoma (7, 88). NAFLD alleviation by flavonoids and their metabolites involves multi-target mechanisms involving the gut-liver axis. They can inhibit the proliferation of pathogenic bacteria while enhancing the abundance of beneficial bacteria such as Bifidobacterium and Lactobacillus, thereby remodeling the gut microbiota. This remodeling helps reduce endotoxin production, increase the conversion of primary bile acids, maintain intestinal immune homeostasis, and improve gut health by enhancing nutrient absorption (89). For instance, quercetin can reverse gut microbiota imbalance, activate relevant gut-liver axis pathways, suppress inflammatory responses, and modulate endoplasmic reticulum stress, consequently alleviating lipid accumulation and associated symptoms (90). Administration of total flavonoids from Ampelopsis grossedentata to NAFLD model mice significantly reduced serum levels of *IL-6* and *TNF-α*, ameliorated hepatic steatosis, decreased the Firmicutes/Bacteroidetes ratio (associated with obesity and metabolic disorders), and increased the abundance of probiotics. This indicates that their beneficial effects on NAFLD are mediated through modulation of the gut microbiota and the “gut-liver axis” (Figure 2) (91). Total flavonoids from *Chrysanthemum morifolium* cv. ‘Chuju’ exert a multi-target synergistic effect via the “gut microbiota-*AMPK/PPAR-α* pathway,” effectively ameliorating high-fat diet-induced lipid metabolism disorders and hepatic injury. This represents a safe and natural dietary intervention strategy for NAFLD with potential application value (92). The gut microbiota plays an irreplaceable and crucial role in human health, making the maintenance of its dynamic balance essential. Given the mutual influence between gut health and liver health, preserving a healthy gut microbiota can help mitigate the onset and progression of NAFLD. In summary, flavonoids can modulate NAFLD through the gut-liver axis and the gut microbiota-*AMPK/PPAR-α* pathway. They help reduce harmful bacteria, increase probiotics, and restore microbial balance. Furthermore, they exert beneficial effects by reducing inflammation and suppressing the systemic diffusion of endotoxin, thereby alleviating NAFLD.

Gut-liver axis modulation by flavonoids is strongly species-dependent. Quercetin and Ampelopsis flavonoids restore microbiota in cats but are limited by obligate carnivore gut structure (10). In dairy cows, citrus flavonoids modify rumen microbiota to reduce endotoxemia (12, 13). In dogs, polyphenols improve dysbiosis and liver enzymes (45). In poultry, chrysanthemum flavonoids reduce Firmicutes/Bacteroidetes ratio and alleviate FLHS via microbiota-*AMPK* crosstalk (14, 93).

## 8 Translational perspectives in veterinary practice

The multifaceted mechanisms of flavonoids described in this review hold significant promise not only for human health but also for veterinary medicine. Given the growing burden of metabolic liver disorders in animals, there is a clear need to translate these mechanistic insights into species-specific applications. For instance, testing flavonoid-rich traditional Chinese medicine (TCM) formulations in clinically relevant models, such as obese cats with hepatic lipidosis or periparturient dairy cows prone to fatty liver, could pave the way for practical phytogenic or nutraceutical strategies (94, 95). Importantly, successful translation will depend on more than just efficacy. Rigorous evaluation of safety, species-specific pharmacokinetics, and delivery formats, like feed-compatible additives for livestock or palatable oral preparations for companion animals, must be prioritized to ensure real-world feasibility. Encouragingly, early studies already support this direction. Quercetin (Q) administered via the duodenum has the potential to improve liver fat deposition in postpartum dairy cows. However, the specific mechanism of this effect still requires further investigation (96). *Ginkgo biloba* extract (GBE) can effectively ameliorate high-fat diet (HFD)-induced FLHS in laying hens by reshaping the gut microbiota (14). In dogs, polyphenol-enriched diets have led to measurable improvements in liver enzyme levels and systemic redox status in obesity-related hepatopathy (97). Together, these findings demonstrate that flavonoids can exert consistent hepatoprotective effects across diverse animal species, reinforcing their potential as both preventive and therapeutic tools for managing NAFLD-like conditions in veterinary practice.

## 9 Multi-target synergistic network: interplay between pathways

While the individual effects of flavonoids on lipid metabolism, oxidative stress, and inflammation are well-documented, the true therapeutic efficacy arises from the intricate crosstalk and positive feedback loops between these targets. The synergy does not merely result from the sum of isolated actions but from a coordinated biological network where the modulation of one pathway potentiates the effect on another.

### 9.1 Antioxidant defense as the foundation for anti-inflammatory action

A pivotal synergistic mechanism lies in the interdependence of redox balance and inflammatory signaling. Oxidative stress is a primary trigger for the activation of the *NF-κB* and *NLRP3* inflammasome pathways. Flavonoids, such as Hesperetin and Quercetin, exert a dual effect: they directly scavenge ROS and upregulate endogenous antioxidants (SOD, CAT) via the *Keap1-Nrf2* pathway. This reduction in oxidative burden subsequently inhibits the *ROS/TXNIP/NLRP3* axis, preventing the cleavage of *pro-IL-1β* and *pro-IL-18* into their active forms. *In vivo* evidence suggests that the anti-inflammatory efficacy of flavonoids is often contingent upon their prior or concurrent antioxidant activity, effectively breaking the vicious cycle where inflammation generates more ROS and vice versa (38, 42, 51).

### 9.2 Metabolic reprogramming suppresses ferroptosis and apoptosis

The activation of *AMPK/SIRT1* signaling by flavonoids serves as a metabolic master switch that intersects with cell survival pathways. By promoting fatty acid *β*-oxidation and inhibiting lipogenesis (via *SREBP-1c*), flavonoids reduce the accumulation of lipid substrates that are prone to peroxidation. This is crucial because lipid peroxidation is the hallmark of ferroptosis. Studies indicate that Kaempferol and Puerarin inhibit ferroptosis not only by chelating iron but also by activating *AMPK*, which enhances mitochondrial function and reduces the lipid peroxide load. Furthermore, improved insulin sensitivity and reduced lipotoxicity decrease the activation of the mitochondrial apoptosis pathway, demonstrating how metabolic correction directly promotes hepatocyte survival (21, 76, 85).

### 9.3 Gut-liver axis: microbial modulation amplifies hepatic protection

The synergy extends beyond the hepatocyte to the gut-liver axis. Flavonoids reshape the gut microbiota, increasing the abundance of beneficial bacteria (e.g., Lactobacillus) that produce short-chain fatty acids (SCFAs). These SCFAs enhance intestinal barrier integrity, reducing endotoxin (LPS) translocation. The reduction in portal LPS levels directly downregulates the *TLR4/NF-κB* pathway in Kupffer cells, thereby suppressing hepatic inflammation. This represents a systemic synergy where modulation of the gut microbiome acts as an upstream amplifier for the direct anti-inflammatory effects of flavonoids within the liver (61, 90).

### 9.4 Integrated signaling hubs: the case of *PI3K/Akt*

The *PI3K/Akt* pathway acts as a central signaling hub that integrates metabolic, inflammatory, and survival signals. Flavonoids like Silibinin and Trilobatin modulate this pathway to simultaneously improve insulin resistance (metabolism), inhibit *NF-κB* nuclear translocation (inflammation), and phosphorylate downstream targets like *mTOR* (autophagy) and *GSK-3β* (apoptosis). This concurrent regulation ensures that while lipid metabolism is being corrected, the cell is also being protected from inflammatory damage and programmed death (52, 65).

## 10 Summary and outlook

**Figure 3:**
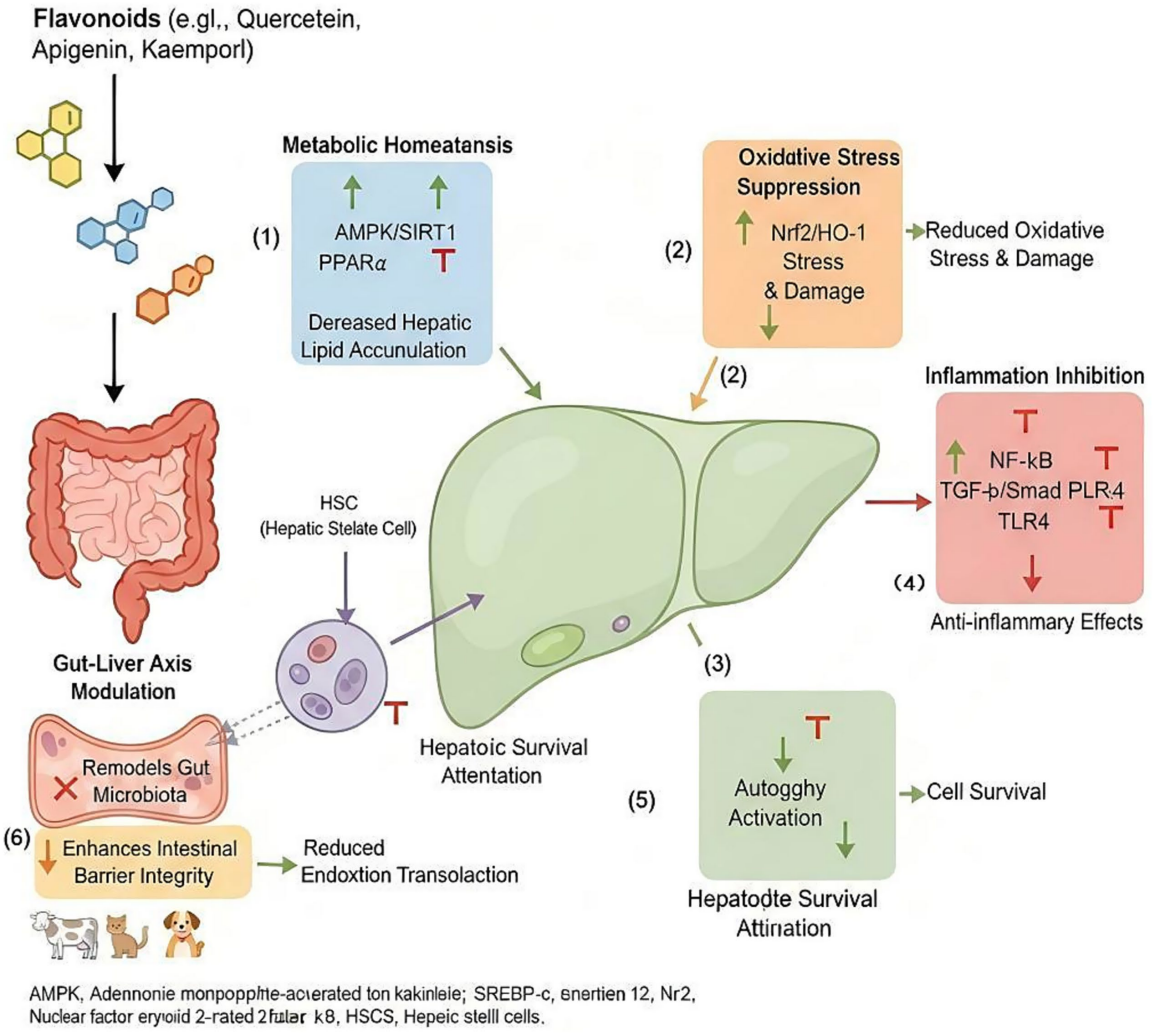
Multi-target mechanisms of flavonoids in the intervention of NAFLD. (1) Metabolic homeostasis (AMPK/SIRT1 and PPAR*α* activation, SREBP-1c inhibition) to alleviate hepatic lipid accumulation in periparturient dairy cows and broiler chickens (2) oxidative stress suppression via Nrf2/HO-1 pathway activation to reduce hepatocellular damage in feline hepatic lipidosis and canine obesity-related liver injury. (3) Inflammation inhibition through NF-κB/NLRP3 and TLR4 pathway downregulation. (4) Hepatic fibrosis attenuation by suppressing HSCs activation and TGF-β/Smad pathway. (5) Hepatocyte survival promotion via ferroptosis/apoptosis inhibition and autophagy activation (6) gut-liver axis modulation by remodeling gut microbiota and enhancing intestinal barrier integrity.

With socio-economic development and changes in lifestyle, the prevalence of NAFLD continues to rise, posing a serious threat to human health and quality of life. Currently, there is a strong demand for natural therapeutics that are both effective and non-resistant. Traditional Chinese medicine flavonoids demonstrate promising multi-target intervention potential in the prevention and treatment of NAFLD. Multiple pathways, including regulating lipid metabolism, alleviating oxidative stress and inflammatory responses, inhibiting the progression of hepatic fibrosis, modulating hepatocyte fate, and remodeling the gut microbiota-flavonoids exhibit significant multi-target potential in NAFLD management. These effects are interwoven and synergistic, collectively forming a complex regulatory network. Specifically, these compounds act through coordinated mechanisms across several dimensions: at the metabolic level, they activate pathways such as *AMPK/SIRT1* and *PPARα*, while inhibiting the *SREBP-1c* and *PI3K/akt* pathways, and promote fatty acid *β*-oxidation, thereby achieving precise regulation of hepatic metabolism. In terms of antioxidant and anti-inflammatory effects, they activate the *Nrf2/HO-1* antioxidant signaling pathway, inhibit inflammatory pathways including *NF-κB* and *TLR4*, and regulate macrophage M1-M2 polarization, effectively mitigating oxidative stress and inflammation. Regarding anti-fibrotic and anti-apoptotic actions, they suppress the *TGF-β/Smad* pathway, hepatic stellate cell activation, and extracellular matrix deposition, modulate apoptosis-related proteins such as *Caspase-3* and *Bcl-2*, and regulate autophagy/ferroptosis processes to enhance cell survival. Additionally, flavonoids can regulate gut microbiota balance and bile acid metabolism, reduce endotoxin release, and restore intestinal barrier integrity, thereby maintaining homeostasis of the gut-liver axis. The above multi-target regulatory effects collectively alleviate hepatic steatosis, reduce inflammation and oxidative stress, slow the progression of liver fibrosis, enhance hepatocyte survival, and improve systemic metabolic health. These interconnected mechanisms form the pharmacological basis for flavonoids in preventing and treating NAFLD, ultimately helping to correct metabolic imbalance and restore health. To visually summarize the systemic mechanisms of flavonoids in NAFLD intervention, integrates these key pathways, systematically illustrating their multi-dimensional and multi-layered pharmacological effects in Figure 3.

However, most current research remains at the stage of cellular and animal studies, with relatively few clinical trials. The low bioavailability of flavonoids limits their clinical application, and the interactions among different flavonoids as well as their optimal combination ratios are not yet clear. Future research should focus on: conducting high-quality clinical studies to evaluate the biosafety and cytotoxicity of flavonoid compounds in NAFLD patients, including long-term monitoring of liver and kidney function. Exploring and developing novel drug delivery systems to enable precise targeted delivery, reduce drug resistance and side effects. We can also use some investigating methods such as polyploid induction in medicinal plants to enhance the production and bioavailability of flavonoids. Delving deeper into the synergistic mechanisms of flavonoids to identify optimal combination strategies and utilizing systems biology and network pharmacology approaches to screen suitable therapeutic targets and comprehensively elucidate the action network of flavonoids in NAFLD prevention and treatment.

## Data Availability

The original contributions presented in the study are included in the article/supplementary material, further inquiries can be directed to the corresponding author.

## Glossary

NAFLD: Non-alcoholic fatty liver disease
AMPK/SIRT1: Adenosine monophosphate-activated protein kinase/Sirtuin 1
PPAR*α*: Peroxisome proliferator-activated receptor α
SREBP-1c: Sterol regulatory element-binding protein 1c
Nrf2/HO-1: Nuclear factor erythroid 2-related factor 2/Heme oxygenase 1
NF-κB/NLRP3: Nuclear factor κB/Nucleotide-binding oligomerization domain-like receptor protein 3
TLR4: Toll-like receptor 4
NASH: Nonalcoholic steatohepatitis
TG: Triglyceride
HCC: Hepatocellular Carcinoma
OCA: Obeticholic Acid
FXR1/TGR5: Farnesoid X receptor 1/Takeda G protein-coupled receptor 5
IRE1a/XBP1s: Inositol-requiring enzyme 1*α*/X-box binding protein 1 spliced
EGCG: Epigallocatechin gallate
FFA: Free Fatty Acid
Acc: Acetyl-CoA Carboxylase
Sirtuin1/AMPK(Sirt1/AMPK): Sirtuin 1/Adenosine monophosphate-activated protein kinase
KMP: Kaempferol
HepG2: Hepatocellular carcinoma cell line
PON1, PPAR-α: Paraoxonase 1, Peroxisome Proliferator-Activated Receptor α
Fdft1, FASN, PPAR-*γ*: Farnesyl-Diphosphate Farnesyltransferase 1, Fatty Acid Synthase, Peroxisome Proliferator-Activated Receptor γ
Pgc1α: Peroxisome Proliferator-Activated Receptor Gamma Coactivator 1-Alpha
LXR: Liver X Receptor
G6Pase: Glucose-6-Phosphatase
PEPCK: Phosphoenolpyruvate Carboxykinase
PI3K/akt: Phosphatidylinositol 3-kinase/Protein kinase B
ROS: Reactive Oxygen Species
RNS: Reactive Nitrogen Species
ETC: Electron Transport Chain
MDA: Malondialdehyde
SOD: Superoxide Dismutase
CAT: Catalase
TFBP: Total Flavonoids of *Broussonetia Papyrifera* Leaves
ANF: Alpha-Naphthoflavone
GSH: Glutathione
T-SOD: Total Superoxide Dismutase
GSH-Px: Glutathione Peroxidase
MAPKs: Mitogen-Activated Protein Kinases
PTFC: Total Flavonoids of Pomelo Peel
Nrf2-ARE: Nuclear Factor Erythroid 2-Related Factor 2-Antioxidant Response Element
TXNIP: Thioredoxin-Interacting Protein
OA: Oleic Acid
Nrf2: Nuclear factor erythroid 2-related factor 2
HFD: High-Fat Diet
STZ: Streptozotocin
ALT: Alanine Aminotransferase
Caspase-1: Caspase-1
IL-1β: Interleukin-1 Beta
IL-18: Interleukin-18
GSDMD: Gasdermin D
mTOR: mammalian Target Of Rapamycin
MCD: Methionine-Choline Deficient
ASC: Apoptosis-Associated Speck-Like Protein Containing a CARD
WT: Wild Type
KCs: Kupffer Cells
IL-6: Interleukin-6
TNF-*α*: Tumor Necrosis Factor-Alpha
CD4 + T: CD4-positive T cell
ATM: Ataxia Telangiectasia Mutated
Ly6Cki: lymphocyte antigen 6 complex, locus C high-expression
TFCTLL: Total Flavonoids from *Carthamus tinctorius* L. Leaf
HSCs: Hepatic Stellate Cells
AST: Aspartate Aminotransferase
HDL-C: High-Density Lipoprotein Cholesterol
Arg-1: Arginase-1
TGF-*β*: Transforming Growth Factor-Beta
UPLC-Q-TOF/MS: Ultra-Performance Liquid Chromatography-High Resolution Mass Spectrometry
ECM: Extracellular Matrix
MMP-2: Matrix Metalloproteinase-2
TIMP-2: Tissue Inhibitor of Metalloproteinase-2
Wog: Wogonin
TGF-β1/Smad3: transforming growth factor-beta/Smad3
p-ERK1/2: phosphorylated Extracellular Signal-Regulated Kinase 1/2
Bcl-XL: B-cell lymphoma-extra large
Col-IV: Collagen Type IV
PC-III: Procollagen Type III
HAase: Hyaluronidase
LN: Laminin
GB: Ginkgolide B
Keap1-NRF2: Kelch-Like ECH-Associated Protein 1-Nuclear Factor Erythroid 2-Related Factor 2
TFMD: Total Flavonoids of Melastoma Dodecandrum Lour
AA: Arachidonic Acid
GPT: Glutamate Pyruvate Transaminase
GOT: Glutamate Oxaloacetate Transaminase
DMY: Dihydromyricetin
L02: Normal human liver cell line
JNK: c-Jun N-Terminal Kinase
AFB1: Aflatoxin B1
PERK: Protein Kinase R-Like Endoplasmic Reticulum Kinase
eIF2α: Eukaryotic Initiation Factor 2-Alpha
3-MA: 3-Methyladenine
